# Mean Atd Angle among Type 2 Diabetes Mellitus Patients Visiting a Tertiary Care Centre: A Descriptive Cross-sectional Study

**DOI:** 10.31729/jnma.8035

**Published:** 2023-02-28

**Authors:** Sheprala Shrestha, Dil Islam Mansur, Rajendra Tamrakar, Pragya Shrestha, Sunima Maskey

**Affiliations:** 1 Department of Anatomy, Kathmandu University School of Medical Sciences, Chaukot, Kavre, Nepal; 2 Department of Internal Medicine, Dhulikhel Hospital, Kathmandu University Hospital, Dhulikhel, Kavre, Nepal

**Keywords:** *dermatoglyphic*, *diabetes mellitus*, *prevalence*

## Abstract

**Introduction:**

An atd angle is one of the dermatoglyphic patterns which is an indication of the degree of distal displacement of the axial triradius on the palm. This is one of the markers of diabetes mellitus that is used as a screening tool in order to reduce the risk of onset and initiate early treatment. The aim of this study is to find the mean atd angle among type 2 diabetes mellitus patients visiting a tertiary care centre.

**Methods:**

This descriptive cross-sectional study was done among diabetic patients in a tertiary centre from 9 June 2021 to 5 May 2022. Ethical approval was taken from Institutional Review Committee (Reference number: KUSMS/IRC 40/2021). Both palm prints of study subjects were taken and the atd angle was measured. Convenience sampling was done. Point estimate and 95% confidence interval were calculated.

**Results:**

Among 133 palm prints of diabetic patients, the mean atd angle was 42.13±4.73° (male: 41.90±4.75° and female: 42.35±4.70°). The right palms showed mean atd angle of 42.31±4.42° and that of left palms was 41.94±5.04°.

**Conclusions:**

The mean atd angle among type 2 diabetes mellitus patients is similar to other studies done in similar settings.

## INTRODUCTION

Dermatoglyphic is the study of specific patterns of epidermal ridges and their configurations on fingers, palms and soles.^1^ Each pattern is unique, having potential to speculate various genetic and acquired disorders with a genetic influence.^2^ An axial triradius (Atd) angle is one of the dermatoglyphic pattern which is an indication of degree of distal displacement of axial triradius on palm.^2^

Type 2 diabetes mellitus (T2DM) has a strong hereditary basis and family history are significant risk factors.^3^ Hence the co-relation between dermatoglyphic and diabetes mellitus.^4^ The atd angle is the most important pattern used as one of the markers for diabetes mellitus that can help in the early identification of at risk individual to develop diabetes mellitus.^5^

The aim of this study is to find the mean atd angle among type 2 diabetes mellitus patients visiting a tertiary care centre.

## METHODS

A descriptive cross-sectional study was performed in the Kathmandu University School of Medical Sciences from 9 June 2021 to 5 May 2022. The data was collected after ethical approval from the Institutional Review Committee (Reference number: KUSMS/IRC 40/2021). Data was collected from dermatoglyphic prints of T2DM patients visiting the Outpatient Department of Internal Medicine, Dhulikhel Hospital. The prints with clean and clear patterns were included in this study. Too faint or bold hand prints were eliminated. Patients with clinically diagnosed congenital anomalies, neurological disorders, carcinomas and psychiatric diseases or any deformity, burn, cut, inflammation and scarred hands were also excluded. Convenience sampling was used. The sample size was calculated using the following formula:


$$\begin{array}{ll} \text{n} & ={\text{Z}}^{2}\times \frac{\sigma^{2}}{{\text{e}}^{\text{2}}}\\ & =1.96^{2}\times \frac{7.81}{2^{2}}\\ & =59 \end{array}$$

Where,

n = minimum required sample size
Z = 1.96 at 95% Confidence Interval (CI)
σ = standard deviation taken as 7.81 from published literature^6^
e = margin of error, 2%

The calculated sample size was 59. On doubling the sample size, the total sample was 118. However, 133 type 2 diabetic mellitus patients were involved. The study subjects were informed about the research and its procedure in detail and their written consent was taken to perform the study. The palm prints were taken by the 'Ink Method' as explained by Cummins and Mildo.^7^ The patients were asked to wash their hands with soap water and dry with a soft cotton cloth to remove any oil or dirt. The duplicating ink was smeared on the both palms uniformly with the help of rubber roller. The hollow area of the palm and flexor creases of the wrist was uniformly inked. The patient's palms were placed on the A4 paper from proximal to distal end one by one. The palm was gently pressed between intermetacarpal grooves at the root of fingers and on the dorsal side corresponding to the thenar and hypothenar regions. The palm was then lifted from the paper in reverse order (distal to proximal end). The printed sheet was coded with name, age, sex, family history, side (right or left palm) and contact number. The atd angle is an indication of the degree of distal displacement of axial triradius. Digital triradius (a), axial triradius (t) and digital triradius (d) were observed with the help of magnifying hand lens and a sharp needle. These triradius were marked with pencil. The angle is then formed by lines drawn from a to t and to d. This atd angle was measured using protractor.

The data were tabulated and entered in Microsoft Excel 2013 and the statistical analysis was done in IBM SPSS Statistics version 23.0. Point estimate and 95% CI were calculated.

## RESULTS

The mean atd angle of the T2DM patients was 42.13±4.73°. Out of total subjects, males were 70 (54.88%) and females were 63 (47.36%) (Table 1).

**Table 1:** Mean atd angle among total subjects according to gender and side wise.

Parameters	Ath angle (Meam±SD)
Male	41.90±4.75°
Female	42.35±4.70°

The mean atd angle of right side was 42.31±4.42° (Table 2).

**Table 2:** Mean atd angle among total subjects according to side.

Side	Mean±SD
Right	42.31±4.42°
Left	41.94±5.04°

The range of this angle of T2DM patients among males is 32-56° and 29-52°among females, whereas right hand ranged 32-56° and 29-55° on left. Moreover, the minimum angle measured 29° on left palm of female whereas maximum was 56° on right palm of male (Table 3).

**Table 3:** Atd angle according to gender and side.

Ath angle	Side	Minimum angle	Maximum angle	Mean±SD
Male	Right	32°	56°	42.03±4.55°
	Left	32°	55°	41.77±4.96°
Female	Right	33°	42°	42.60±4.28°
	Left	29°	52°	42.10°±5.11°

## DISCUSSION

As early as 300 B.C., dermatoglyphic was used as identity verification of individual person in China, as early as 702 A.D. in Japan, and since 1902 in United States.^8^ In 1926, Cummins and Midlo were the first to coin the term "Dermatoglyphics" (from two Greek words-derma: skin, glyphe: carving) but these patterns develop in early fetal life and are genetically determined was done by Schaumann and Alter in 1976.^9^ Dermatoglyphic patterns with clinically diagnosed diabetes mellitus was first studied among a group of Russian children.^10^

In the present study, the atd angle is observed to be 42.13°±4.73° which is larger among T2DM patients as compared to normal individuals.^11^ The mean atd angle of T2DM patients in the present study was alike most of the studies done in India.^12-16^ However, some researchers reported a greatly broader atd angle with maximum of 80°, which is much larger than the present study findings.^17-19^ In addition, only a handful of study showed narrow angle than the present study.^20^ This angle tends to decrease with age as the palm grows more in length than in breadth. Also, the size of this angle is affected by the amount of spreading of fingers when print is taken and also by the pressure exerted while the palm is printed.^7^ The aforesaid basis could justify the dissimilarity of the atd angle in the different studies.

The mean atd angle of T2DM patients is larger in females than among males in the present study, which is in concord with majority of researches.^12-14^ The right hand of T2DM patients presented greater atd angle than on the left hand in the present study. Only a study showed similarity with the present study.^14^ While previous studies reported the angle to be larger on the left hand of T2DM patient.^17,18^ On the other hand, many studies stated similar angle regardless of side of hand.^12,13,15,16^ The atd angle of the both hand is observed to be greater in females than males in the present study which agrees with the prior researches.^13,14^

This study has certain limitations. Primarily, the study was confined to the T2DM patients visiting Dhulikhel Hospital and thus could not be a representative study of the whole country. Besides this, the sample size was limited with unequal distribution of gender, side and as well as age, ethnic and race which possibly will affect the comparison of values. The atd angle gets affected even by pressure exerted on the palm when print was taken. So, using software digital devices could yield better outcome. The study has also not considered the other aspects of dermatoglyphics like thenar patterns, hypothenar patterns and finger print patterns. Moreover, other factors like occupation and lifestyle also influence the dermatoglyphics. Further studies with a large sample size and other relevant factors correction including the different geographical regions of Nepal need to be performed.

## CONCLUSIONS

The mean atd angle is similar to other studies done in similar settings. Knowledge of dermatoglyphic patterns of T2DM patients will aid the clinicians as an economical and preliminary tool to screen its onset, in consequence to refine the result of treatment and prevent further complications.

## References

[1] Cummins H, Midlo C (1961). Finger Prints Palms and Soles - An Introduction to Dermatoglyphics..

[2] Miller JR (1973). Dermatoglyphics.. J Invest Dermatol..

[3] Jameson JL, Fauci AS, Kasper DL, Hauser SL, Longo DL, Loscalzo J (2017). Harrison's Principles of Internal Medicine..

[4] Sudagar M, Radha K, Duraipandian K, Sundaravadhanam KVK (2014). Study of palmar patterns in diabetic patients.. International Journal of Advances in Medicine..

[5] Park K (2021). Park's textbook of preventive and social medicine..

[6] Sharma MK, Sharma H (2012). Dermatoglyphics - A diagnostic tool to predict diabetes.. J Clin Diagn Res..

[7] Cummins H, Midlo C (1943). Finger Prints of Palms and Soles - An Introduction to Dermatoglyphics..

[8] Mulvihill JJ, Smith DW (1969). The genesis of dermatoglyphics.. J Pediatr..

[9] Schaumann B, Alter M (1976). Dermatoglyphics in medical disorders..

[10] Bets LV, Dzhanibekova IV, Lebedev NB, Kuraeva TL (1994). Constitutional and dermatoglyphic characteristics of children with diabetes mellitus]. Probl Endokrinol (Mosk)..

[11] Gautam M, Singh UP (2017). Palmar dermatoglyphics of the khatiks and kumbhars of Lucknow, Uttar Pradesh.. Asian Man (The) - An International Journal..

[12] Srivatsava S, Burli S (2019). A study of palmar dermatoglyphics in type 2 diabetes mellitus in a Bangalore based population.. Indian J Clin Anat Physiol..

[13] Bala A, Deswal A, Sarmah PC, Khandalwal B, Tamang BK (2015). Palmar dermatoglyphics patterns in diabetes mellitus and diabetic with hypertension patients in Gangtok region.. Int J Adv Res..

[14] Sharma MK, Sharma H (2012). Dermatoglyphics - A diagnostic tool to predict diabetes.. J Clin Diagn Res..

[15] Shirahatti A, Dixit D (2019). A cross - sectional study of palmar dermatoglyphic pattern in patients with type 2 diabetes mellitus at a hospital in South India.. Indian J Clin Anat Physiol..

[16] Mittal M, Lala BS (2013). Dermatoglyphics-an economical tool for prediction of diabetes mellitus.. International Journal of Medical Health Sciences..

[17] Nayak V, Shrivastava U, Kumar S, Balkund K (2015). Dermatoglyphic study of diabetes mellitus type 2 in maharastrian population.. International Journal of Medical Science Research and Practice..

[18] Tarigoppula RK VN, Manasa S, Raheem AMB, Patil Sk (2018). Evaluation of the role of dermatoglyphics, Oral micronuclei and ABO blood grouping in determining Type 2 Diabetes- A multi-parameter Approach.. J Clin Diagnostic Res..

[19] Ojha P, Gupta G (2014). Dermatoglyphic study - a comparison in hands of type II diabetes mellitus patients and normal persons of Udaipur region.. J Evol Med Dent Sci..

[20] Rajanigandha V, Mangala P, Latha P, Vasudha S (2006). Digito-palmar complex in non-insulin dependent diabetes mellitus.. Turk J Med Sci.

